# LAMC2 regulates the proliferation, invasion, and metastasis of gastric cancer via PI3K/Akt signaling pathway

**DOI:** 10.1007/s00432-024-05720-7

**Published:** 2024-05-04

**Authors:** Lulu Cheng, Xiaofei Li, Wenhui Dong, Jing Yang, Pengmei Li, Xihui Qiang, Jiajun Yin, Lianyi Guo

**Affiliations:** 1 Department of Gastroenterology, The First Affiliated Hospital of Jinzhou Medical University, Jinzhou, 121001 China; 2 Department of Pathology, The First Affiliated Hospital of Jinzhou Medical University, Jinzhou, 121001 China; 3 Department of General Surgery, Affiliated Zhongshan Hospital of Dalian University, Dalian, 116001 China

**Keywords:** LAMC2, PI3K/AKT signaling pathway, EMT, Gastric cancer

## Abstract

**Objectives:**

Gastric cancer (GC) is a prevalent malignant tumor widely distributed globally, exhibiting elevated incidence and fatality rates. The gene LAMC2 encodes the laminin subunit gamma-2 chain and is found specifically in the basement membrane of epithelial cells. Its expression is aberrant in multiple types of malignant tumors. This research elucidated a link between LAMC2 and the clinical characteristics of GC and investigated the potential involvement of LAMC2 in GC proliferation and advancement.

**Materials and methods:**

LAMC2 expressions were detected in GC cell lines and normal gastric epithelial cell lines via qRT-PCR. Silencing and overexpression of the LAMC2 were conducted by lentiviral transfection. A xenograft mouse model was also developed for in vivo analysis. Cell functional assays were conducted to elucidate the involvement of LAMC2 in cell growth, migration, and penetration. Further, immunoblotting was conducted to investigate the impact of LAMC2 on the activation of signal pathways after lentiviral transfection.

**Results:**

In the findings, LAMC2 expression was markedly upregulated in GC cell lines as opposed to normal gastric epithelial cells. In vitro analysis showed that sh*-*LAMC2 substantially inhibited GC cell growth, migration, and invasion, while oe*-*LAMC2 displayed a contrasting effect. Xenograft tumor models demonstrated that oe-LAMC2 accelerated tumor growth via high expression of Ki-67. Immunoblotting analysis revealed a substantial decrease in various signaling pathway proteins, PI3K, p-Akt, and Vimentin levels upon LAMC2 knockdown, followed by increased E-cadherin expression. Conversely, its overexpression exhibited contrasting effects. Besides, epithelial-mesenchymal transition (EMT) was accelerated by LAMC2.

**Conclusion:**

This study provides evidence indicating that LAMC2, by stimulating signaling pathways, facilitated EMT and stimulated the progression of GC cells in laboratory settings and mouse models. Research also explored that the abnormal LAMC2 expression acts as a biomarker for GC.

**Supplementary Information:**

The online version contains supplementary material available at 10.1007/s00432-024-05720-7.

## Introduction

GC is a prevalent and invasive malignant tumor affecting the digestive system. It was identified as the second major reason for cancer-related deaths globally (Ferlay et al. 2019). According to the most recent cancer records in the United States, the incidence of GC is projected to reach approximately 26,380 new cases in 2022. Further, it is anticipated that the mortality rate associated with this malignancy may result in more than 11,090 deaths (Siegel et al. 2022). Studies show that GC has the highest incidence and mortality rates in East Asian regions, including China, Japan, Singapore, and South Korea (Fitzmaurice et al. 2019; Rahman et al. 2014). The incidence exhibits a higher prevalence among the male population than the females. China has been identified with a high incidence of GC. However, the lack of timely diagnosis and treatment results in many patients being diagnosed at a severe stage. Consequently, the chance of surviving five years for these patients is less than 30%. The postoperative recurrence and dissemination are the primary contributing factors for the reduced survival rate (Joshi et al. 2021). GC is an important concern in global health, necessitating the exploration and inspection of specific molecules and mechanisms linked with its metastatic progression. This is necessary for the development of effective diagnostic and therapeutic strategies.

Laminin, a glycoprotein, comprises three polypeptide chains (*α*, *β*, and *γ*) linked by three disulfide bonds. In 1979, it was initially isolated from transplantable tumors (EHS sarcoma) and basement membrane-producing cells in mice (Chung et al. 1979; Timpl et al. 1979). Laminin is an extracellular matrix (ECM) protein involved in multiple biological processes, including cellular growth, maintaining cell phenotype, cellular adhesion, migration, and differentiation (Domogatskaya et al. 2012). LAMC2 is responsible for encoding the gamma-2 chain subunit of laminin, one of the three subunits comprising LM-332. It is synthesized and released by keratinocytes in humans. Based on previous studies, LAMC2 has been implicated in the invasion and metastasis of various cancer types, such as colorectal, pancreatic, lung, cervical, and liver cancer, among others. However, the precise mechanism by which it exerts its effects on GC is not fully elucidated (Cen et al. 2021; Huang et al. 2021; Huang et al. 2017; Moon et al. 2015; Sentani et al. 2014).

The progression of tumors entails the growth of multiple malignant phenotypes, with metastasis responsible for over 90% of cancer-related mortalities. Regulation of tumor progression involves interactions between various components within the tumor microenvironment, immune cells, stromal cells, chemokines, and tumor cells (Chaffer et al. 2011). EMT entails the conversion of epithelial cells into a mesenchymal phenotype, which is not only linked to tumor dissemination but also to their initiation, invasion, and resistance to therapeutic drugs (Pastushenko et al. 2019). In the EMT process, notable alterations manifest in cell morphology, cell–cell interactions, and various signaling pathways. These changes collectively facilitate the invasion of cancer cells into neighboring tissues, disseminating tumors (Pastushenko et al. 2019). Phosphoinositide 3-kinase (PI3K) and protein kinase B (PKB/Akt) represent crucial proteins in the PI3K/Akt signaling cascade, which is controlled by several pathways and has been implicated in the development and advancement of multiple cancer types. The role of activated Akt in regulating the cell cycle and its effect on cancer cell growth, anti-apoptosis, migration, and invasion capabilities has been extensively studied (Bamodu et al. 2020; Jin et al. 2022; Lei et al. 2022; Stanciu et al. 2022). The PI3K/Akt signaling pathway is considered to be one of the core pathways regulating the EMT process. Activation of this pathway promotes the downregulation of epithelial markers, such as E-cadherin, and induces the upregulation of mesenchymal markers, such as vimentin (Verma et al. 2024; Xu et al. 2024). This series of changes constitutes a key step in the EMT process. Additionally, the PI3K/Akt signaling pathway also profoundly affects the rearrangement of the cytoskeleton and the disruption of intercellular connections, further promoting the transition of cells from epithelial to mesenchymal phenotypes (Zhang et al. 2023). Therefore, a thorough exploration of the complex relationship between EMT and PI3K/Akt signaling is expected to provide valuable insights and evidence for the development of cancer treatment strategies. However, the precise role of LAMC2 in advancing and disseminating GC via the PI3K pathway and EMT mechanisms has yet to be fully elucidated.

The precise molecular mechanism underlying the impact of LAMC2 on the biological characteristics of GC remains poorly understood (Xu et al. 2017). Therefore, this study has been conducted to gain further insights into the effects of LAMC2 on GC. An upregulation of LAMC2 in GC was initially noted by analyzing the data from The Cancer Genome Atlas (TCGA) and Gene Expression Omnibus (GEO) databases (Chandrashekar et al. 2017). The functional and mechanistic role of LAMC2 in human GC cell lines AGS and MKN45 was subsequently explored. LAMC2 is substantially involved in human GC cell growth and malignant progression by controlling the PI3K/Akt pathway.

## Materials and methods

### Bioinformatics analysis

The UALCAN database (http://ualcan.path.uab.edu/index.html) represents an easy and interactive web-based platform for examining cancer-related transcriptome data. This study used GC-related chip data and focused on investigating the expression of LAMC2. Box plots were generated to visually represent the expression of LAMC2, which concisely summarize the distribution and variability of LAMC2 level within the GC dataset. This study facilitated the analysis of LAMC2 in GC and normal control from TCGA database.

Transcriptomic data from 375 GC patients were retrieved from TCGA. Data preprocessing was conducted using R version 4.3.0, and the differential pattern level of LAMC2 was monitored via the Limma package. Following the median value of LAMC2 level, GC cells were segregated into two distinct groups: high LAMC2 and low LAMC2 expressions. GC-related microarray data were acquired from the GEO database, specifically, two microarrays (https://www.ncbi.nlm.nih.gov/geo; GSE13911 and GSE118916). A log-fold change ≥ 1 and *p* <  0.05 was deemed the threshold for selecting differentially expressed genes (DEGs). A Venn diagram was constructed to assess the overlap of increased DEGs among the databases: GSE13911, GSE118916, and TCGA.

Interaction genes were retrieved from the STRING (https://string-db.org) database to construct a gene interaction network for the elevated gene level at the DEG intersection from chip and TCGA data (Szklarczyk et al. 2023). Enriched pathways in GC were examined for two sample groups by R programming language and packages such as "ggplot2," "clusterProfiler," and "enrichplot," Gene Ontology (GO) and Kyoto Encyclopedia of Genes and Genomes (KEGG) gene enrichment analysis software. Potential mechanisms of LAMC2 in GC were also explored (Jin et al. 2023).

### Sample collection

To conduct immunohistochemistry investigations, wax blocks of postoperative pathological specimens from 16 paired cases of GC with paracancerous noncancerous tissues were first acquired. Included were seventy patients who had undergone surgical resection for GC at the First Affiliated Hospital of Jinzhou Medical University between 2017 and 2021. Postoperative pathology provided complete clinical information and follow-up data for the diagnosis of GC. The tissues were immersed in paraffin and then transformed into tissue microarrays. Disease-free survival (DFS) and overall survival (OS) were determined as the periods from the initial surgery to the confirmed recurrence or progression of the disease, and death, respectively. The relevant diagnostic reports were also included for the record. All the enrolled GC patients were not treated with preoperative radiotherapy, chemotherapy, or biological targeted and immunotherapy. Tissue samples were collected with the Medical Ethics Committee for Scientific Research approval at the First Affiliated Hospital of Jinzhou Medical University.

### Immunohistochemistry (IHC)

GC and adjacent normal tissues were fixed in formalin and parraffinized for analysis. Following a 2 h incubation at 60 °C, the sections were deparaffinized in xylene and rehydrated with gradually decreased ethanol concentrations. Endogenous peroxidase was blocked after treatment with 3% hydrogen peroxide for 20 min at room temperature (RT). Subsequently, sections were placed in EDTA-Tris for a high-pressure antigen retrieval step of 10 min incubation. Non-specific antigens were blocked at RT for 30 min with 10% normal goat serum. For secondary antibody interaction, sections were incubated at 4 °C overnight with rabbit anti-LAMC2 (Abcam, Cat. No. Ab210959, 1:500) and rabbit anti-Ki67 (Proteintech, Cat No. 27309–1-AP, 1:5000). These sections were subsequently rinsed with PBS and incubated at RT for 20 min utilizing secondary antibodies conjugated to HRP (Proteintech, Cat No. PR30012). Sections were detected using DAB staining, stained with hematoxylin, followed by dehydration in alcohol. Before mounting, sections were cleared with xylene. Following the established protocol, the quantification of protein expression was conducted via immunoreactivity score (IS). The IS was determined by combining the score assigned to staining intensity with the proportion of positive cells. The scoring criteria are outlined as follows: Staining intensity score:0, Negative;1, Weakly positive;2, Slightly positive;3, Strongly positive. Stained cells percentages:0, < 5%;1, 5–25%; 2, 26–50%;3, 51–75%;4,  > 75%. The IS score ranges from 0 to 12:0, Negative; 1–3, Weakly positive; 4–8, Slightly positive; 9–12, Strongly positive (Ji et al. 2024). Samples were divided into high-expression (≥ 4 points) or low-expression (< 4 points) groups based on the LAMC2 expression in each tissue section. Two researchers independently analyzed all visual images of the stained sections randomly.

### Cell culture

This study used differentiated GC epithelial HGC-27, AGS, and MKN45 along with normal gastric mucosal GES-1 cell lines. These cell lines were procured from the Chinese Academy of Sciences Cell Bank. These cells were maintained and incubated in RPMI-1640 medium (Gibco, USA) supplemented with 10% fetal bovine serum, 100 U/mL Penicillin, and 100μg/mL Streptomycin at 37 °C with 5% CO_2_. Cells were displayed with potential adhesion, and subculturing was typically conducted every 2–4 days following their growth characteristics.

### Real-time quantitative polymerase chain reaction (qRT-PCR)

Cells were homogenized for total RNA isolation using TRIzol reagent (Invitrogen, CA, USA). Subsequently, the extracted RNA was used for cDNA synthesis via PrimeScriptTM RT reagent Kit with gDNA Eraser (Takara, Japan). Quantitative PCR was executed with SYBR Premix Ex Taq (Vazyme, Shanghai, China). GAPDH was utilized as a housekeeping gene for normalizing the Ct values of the samples. Data were analyzed by the 2^−ΔΔCt^ method. Data were examined by conducting triplicate experiments. The primer sequences are presented below:

LAMC2-F: 5'GGAATTTGGACAAGTGCTGTTG3'

LAMC2-R: 5'TGACTGGTTGCACTCTGTTCTG3'

GAPDH-F: 5'ACAACTTTGGTATCGTGGAAGG3'

GAPDH-R: 5'GCCATCA CGCCACAGTTTC3'

### Western blotting

Cells were harvested for total protein extraction utilizing the Beyotime Biotechnology Total Protein Extraction Kit. Protein content was quantified using the BCA method. Following denaturation at a high temperature of 10 min, proteins were subsequently loaded into SDS-PAGE. The resulting protein bands were transferred onto a 0.45 µm PVDF membrane (Millipore). These membranes were placed in a blocking solution (5% skim milk) for 2 h. Next, it was incubated overnight at 4 °C with primary antibodies. Following the initial step, secondary antibodies were added to the membrane and incubated at RT for 1 h. Following antibodies were used included anti-LAMC2 (Abcam, ab274376, 1:1000), anti-pAkt (S473) (CST#4060, 1:1000), anti-Akt (CST#9272, 1:1000), anti-E-cadherin (CST#3195, 1:1000), anti-Vimentin (CST#5741, 1:1000), anti-PI3K (CST#4249, 1:1000), and GAPDH (CST#5174, 1:1000). ECL A and B solutions (1:1) were mixed and subsequently added to the membrane. The visualization of protein bands was achieved via a chemiluminescence imaging system. Data were examined by conducting triplicate experiments.

### Virus transfection for lentivirus

This investigation involved the shRNA vector, specifically the pLenti-U6-shRNA-CMV-GFP-2A-Puro, while the control vector was the Lenti-CMV-CBH-GFP-2A-Puro-Blank Virus. The vector used for overexpression in this study was pLenti-GIII-CMV-CBH-GFP-2A-Puro, which exhibited increased target gene expression. In contrast, the control viruses lacked any genetic material, serving as a baseline for comparison. ShRNA interference virus transduction was conducted using MKN45 cells, while overexpression virus transduction was conducted using AGS cells. The virus titer was determined using established MOI (Multiplicity of Infection) from preliminary experiments and the corresponding formula. At the time of infection, the growth medium was replaced with a mixture comprising 5 µg/mL Polybrene. Cell infection was detected via fluorescence microscopy after 72 h incubation. Subsequently, the growth medium was discarded, and a new medium was added with Puromycin for selection. Cells were harvested after 48 h, followed by the execution of Western blot analysis to validate the efficacy of both overexpression and interference (Lv et al. 2021).

## Cell functional assays

### CCK-8 experiment

Cells in the logarithmic growth phase were selected and subsequently cultured into 96-well plates at 2 × 10^3^ cells/well density. Each group was represented by 5 replicate wells using 4 plates to ensure statistical accuracy. Cells were allowed to adhere to standard culture conditions at 24, 48, 72, and 96 h, correspondingly. In each well, 10μL of CCK-8 solution (APExBIO Technology) was added. These well plates were incubated in standard condition for 2 h. Absorbance was taken at 450 nm using a microplate reader, and a cell growth curve was generated accordingly. Data were examined by conducting triplicate experiments.

### Colony formation assay

Cells were cultured into 6-well plates at 1 × 10^3^ cells/well density. Cells were uniformly distributed by gently rocking. After 10 days, colonies were observed. The culture medium was removed, and the colonies were fixed with 4% paraformaldehyde (PFA). Subsequently, the fixed colonies were stained with 0.1% crystal violet. ImageJ software was used for manually quantifying colonies exceeding a threshold of 50. Data were examined by conducting triplicate experiments.

### Scratch assay

Cells were cultured into 6-well plates at 1 × 10^6^ cells/ well. A scratch was induced via a 200μL pipette tip when the cellular monolayer achieved full confluence. The cells were then cultured in an FBS-free medium. The migration of the cells was measured in the Image J software by measuring the distance between the edges of a scratch at 0, 24, and 48 h. The data was examined by conducting triplicate experiments.

### Transwell migration and invasion assay

In this assay, the upper chamber of the Transwell (Corning, USA) was removed, following the manufacturer's instructions. Before the experiment, the Matrigel coating was applied to the upper chamber and incubated for 3 h. In the migration assay, Transwells without Matrigel coating were employed. The upper chamber was loaded with 5 × 10^4^ cells in suspension. In the lower chamber, 600μL of cell culture medium with 10% serum was added to each (24-well plate). Following cell seeding, the culture was allowed to grow for 36 h in a controlled culture environment (Qu et al. 2023). Following incubation, the medium was discarded, and cells were fixed with 4% PFA and stained with a 0.1% crystal violet for 20 min. These cells were washed multiple times with PBS and detached from the upper chamber using a cotton swab. These chambers were allowed for air drying, and microscopic images were captured. To quantify the number or distribution of migrated or invaded cells, at least five distinct fields were randomly selected. The experiment was conducted in triplicate to ensure reproducibility of the results.

### Tumor xenograft model

Twelve BALB/C nude mice, aged between 4 and 5 weeks, were selected as the experimental models. Mice were maintained in a controlled SPF-level environment. The protocol was received after approval from the Ethics Committee of Jinzhou Medical University. Cell suspensions were prepared, followed by microscopic examination of cell viability. Mice were allocated into four groups randomly, and 5 × 10^6^ cells were administered subcutaneously near the armpits of each mouse. Mice were regularly monitored for tumor development. Subsequently, one week after initial observation, the volumes of tumors were noted every 5 days. The formula used to quantify the tumor volume: V = (Length × Width^2^)/2 (Li et al. 2024). Following 35 days, mice were sacrificed, and tissues were harvested, weighed, and analyzed by gross examination. Formalin was used to fix the tissues for subsequent tissue embedding and immunohistochemical analysis.

### Data analysis

Data were analyzed via SPSS 26.0 and GraphPad Prism 9.0 statistical software. A single-sample K–S normality test and Levene's test for homogeneity of variances were conducted initially for continuous variables. Student's *t* test was conducted to compare two datasets that were assumed to follow a normal distribution. In instances where there were disparities in variances, an approximate *t* test was employed. One-way analysis of variance (ANOVA) was performed to assess the disparities among multiple groups. The Pearson chi-square test or Fisher's exact test was utilized to examine the association between LAMC2 and the clinicopathological parameters of the patients. The Kaplan–Meier approach was utilized to examine the impact of LAMC2 on DFS and OS in patients with GC. Analyzed in this study was the impact of aberrant expression of LAMC2 and clinicopathological characteristics on the survival prognosis of patients using a multifactorial COX proportional risk model, which incorporated a single component. Triplicates findings were obtained and presented as "mean ± SEM." A significance level of *p* < 0.05 was marked to be statistically considerable.

## Results

### Positive correlation between LAMC2 and GC development

**Fig. 1:**
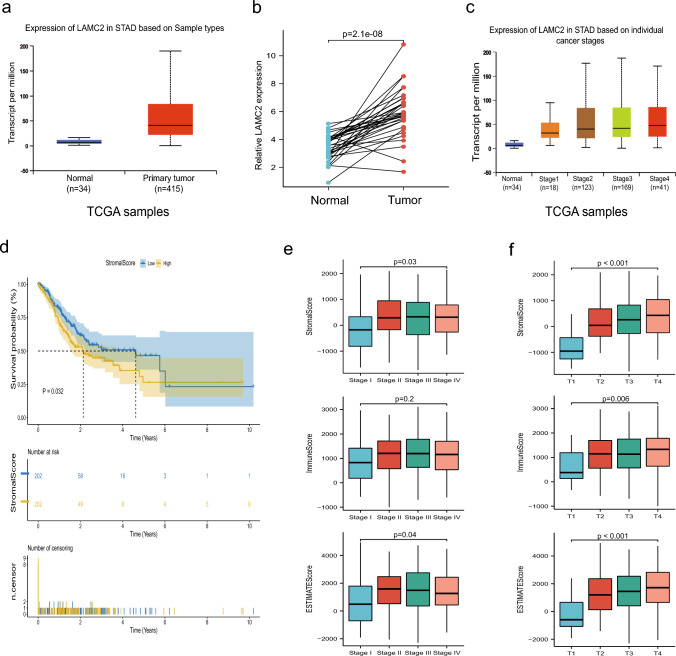
Expression analysis of LAMC2 mRNA in normal and GC tissues as well as the correlation of scores with the survival of patients with STAD. **a**, **b** The levels of LAMC2 mRNA in normal gastric tissues are significantly lower than in GC tissue samples. **c** Expression of LAMC2 in STAD based on individual cancer stages. **d** Kaplan–Meier survival analysis for STAD patients grouped into high or low score in StromalScore determined by the comparison with the median. *p* = 0.032 by log-rank test. **e** Distribution of ImmuneScore, StromalScore, and ESTIMATEScore in stage. *p* = 0.03, 0.2, and 0.04, respectively, by Kruskal–Wallis rank sum test. **f** The distribution of these three types of scores in T classification. (*p* = 0.001, 0.006, 0.001 for ImmuneScore, StromalScore, and EstimateScore, respectively, by Kruskal–Wallis rank sum test)

Following the transcriptomic database, the gene pattern of LAMC2 in GC and adjacent tissues was investigated via the UALCAN database and the R programming language. The findings demonstrated a significant upregulation of LAMC2 expression in GC compared to adjacent tissues. Further, a positive correlation between LAMC2 expression and clinical staging was observed (*p* < 0.001, Fig. 1a–c). The R programming language was utilized to examine the relative abundance of tumor-infiltrating immune cells and the levels of immune and stromal components in samples of STAD. Besides, the ESTIMATE algorithm and Kaplan–Meier survival analysis were also conducted to investigate the impact of the Stromal Score. Figure 1d shows a positive correlation between the proportion of stromal components and overall survival. To explore the link between the proportions of immune and stromal components and clinical pathological features, the relevant clinical data of STAD cases were acquired from the TCGA database. Figure 1e and f demonstrates a positive correlation between the Stromal Score and both individual cancer stages and T-stage. Similarly, the Immune Score and ESTIMATE Score were positively associated with the T-stage. The findings also indicated a potential link between the ratios of immune and stromal constituents and the advancement of STAD, including invasion and metastasis.

**Fig. 2:**
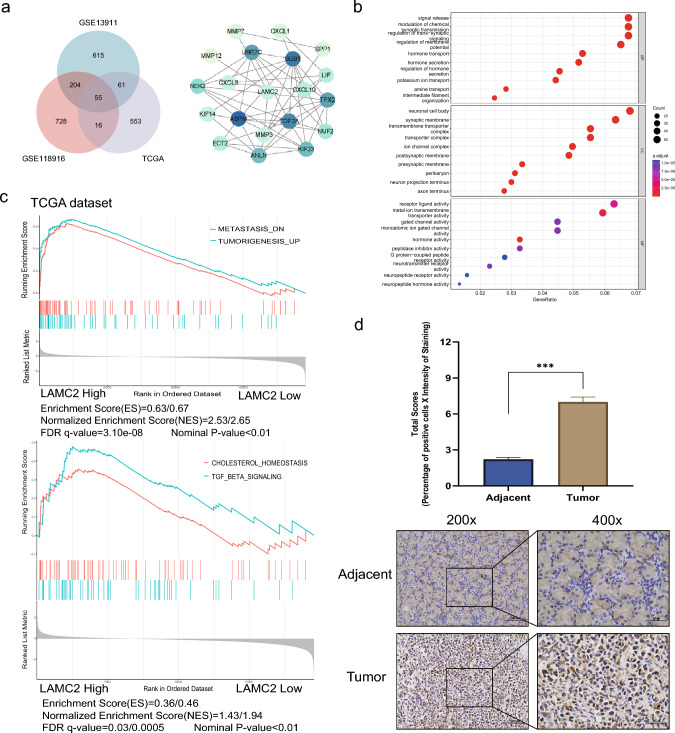
Bioinformatics Enrichment Analysis along with IHC protein expression in human GC tissues. **a** Venn analysis of DEGs in public databases (TCGA, GSE13911, and GSE118916) and STRING protein–protein interaction network reveals the significant prominence of LAMC2. **b** GO analysis of differentially expressed genes. **c** GSEA evaluating LAMC2 expression in GC. **d** IHC analysis shows that LAMC2 protein levels are significantly higher in matched GC compared with the adjacent normal tissue. Representative images at 200 × and 400 × magnification, respectively. (*p < 0.05;**p < 0.01; ***p < 0.001)

Venn and STRING protein–protein interaction analyses were conducted on the DEGs obtained from the GEO database to screen GC-related genes. These findings evaluated the intersections of genes (Fig. 2a) and demonstrated that LAMC2 was present among the shared genes. GO and KEGG enrichment analyses were conducted to understand the biological significance of the DEGs further. GO analysis revealed that LAMC2 was potentially implicated in many biological events, including translation initiation, RNA degradation, regulation of the actin cytoskeleton, and protein targeting to the cell membrane and endoplasmic reticulum. LAMC2 may also be involved in the activation of neutrophils during immune responses (Fig. 2b). Moreover, gene set enrichment analysis (GSEA) was executed to examine a significant link between high LAMC2 expression and various GC-related processes, including the activation and migration of GC cells via phosphatidylinositol signaling system, tumor invasion and metastasis, and EMT mediated by the TGF-β pathway (Fig. 2c).

### Elevated LAMC2 expression in GC tissues and its association with clinical prognosis

To explore the role of LAMC2, immunohistochemistry evaluated the LAMC2 expression in 10 pairs of GC and adjacent tissues. Findings demonstrated that LAMC2 was primarily localized in the cytoplasm of human GC cells. Its expression in GC tissues was substantially upregulated compared to normal tissues (*p* < 0.05), consistent with the database analysis. HE analysis revealed the presence of nuclear atypia, characterized by abnormal nuclear morphology. Additionally, a high ratio of nucleus-to-cytoplasm was observed in the tumor cells, indicating an altered cellular composition. Further, occasional mitotic figures, indicative of cell division, were identified within the tumor cells. Focal necrosis covering one-quarter of the area revealed distinct features, including nuclear fragmentation or dissolution and intensified acidophilia. IHC analysis exhibited markedly enhanced staining intensity of LAMC2 in GC tumor tissues (*p* < 0.001, Fig. 2d).

To examine the relationship between the level of LAMC2 expression and clinicopathological characteristics, we conducted a chi-square test on the tissue microarrays obtained from 70 patients with GC. Our investigation revealed a significant association between elevated expression of LAMC2 and T stage (*p* = 0.001), N stage (*p* = 0.048), M stage (*p* = 0.011) and histological differentiation (*p* = 0.016). However, no correlation was detected between LAMC2 expression and other clinicopathologic parameters (Table 1). According to our findings in the GEPIA2.0 database (http://gepia2.cancer-pku.cn), there was no correlation between the expression of LAMC2 and overall survival in patients with GC (FigureS1.a). Surprisingly we discovered a strong correlation between the expression level of LAMC2 and disease-free survival (DFS) in patients with GC. Specifically, individuals with high LAMC2 expression exhibited a shorter period of being free from illness and a more unfavorable prognosis compared to those with low expression (*p* < 0.05, FigureS1b).

**Table 1:** The relationship between LAMC2 expression and clinicopathological characteristics of GC

Characteristics	LAMC2	Expression	χ^**2**^	P value
	Low(*n* = 27)	High(*n* = 43)		
*Gender*			1.653	0.199
Male	22 (31.4%)	29 (41.4%)		
Female	5 (7.1%)	14 (20%)		
*Age (years)*			1.079	0.299
≥ 65	11 (15.7%)	23 (32.9%)		
< 65	16 (22.9%)	20 (28.6%)		
*Tumor size (cm)*			1.248	0.264
< 5	15 (21.4%)	18 (25.7%)		
≥ 5	12 (17.1%)	25 (35.7%)		
*Differentiation*			5.762	**0.016**
Poor	9 (12.9%)	27 (38.6%)		
Well, moderate	18 (25.7%)	16 (22.9%)		
*T stage*			16.116	**0.001**
T1	10 (14.3%)	4 (5.7%)		
T2	9 (12.9%)	6 (8.6%)		
T3	6 (8.6%)	19 (27.1%)		
T4	2 (2.9%)	14 (20%)		
*N stage*			7.904	**0.048**
N0	7 (10%)	5 (7.1%)		
N1	11 (15.7%)	9 (12.9%)		
N2	4 (5.7%)	11 (15.7%)		
N3	5 (7.1%)	18 (25.7%)		
*M stage*			6.425	**0.011**
M0	26 (37.1%)	31 (44.3%)		
M1	1 (1.4%)	12 (17.1%)		
*Vascular invasion*			0.814	0.367
Negative	9 (12.9%)	19 (27.1%)		
Positive	18 (25.7%)	24 (34.3%)		
*Lymph node metastasis*			1.334	0.248
Negative	10 (14.3%)	22 (31.4%)		
Positive	17 (24.3%)	21 (30%)		

Ultimately, the COX risk regression model was utilized to perform both unifactorial and multifactorial survival analysis of DFS in patients with GC. In the unifactorial analysis, the T stage, tumor recurrence, and LAMC2 expression were found to be significantly associated with DFS (Table 2). Furthermore, we incorporated independent prognostic factors identified in the univariate analysis of DFS into the multivariate analysis. The findings revealed that the expression of LAMC2 remained a significant independent prognostic factor for DFS in patients with GC. The data indicate that LAMC2 is highly expressed in GC tissues and could serve as an independent predictive biomarker for DFS following radical surgery in patients with GC.

**Table 2:** Univariate and multivariate Cox professional hazard models for DFS after surgery

Characteristics	Univariate analysis		Multivariate analysis	
	Hazard ratio (95% CI)	*P* value	Hazard ratio (95% CI)	*P* value
Gender (Male vs. Female)	1.731 (0.891–3.362)	0.105		
Age (≥ 65 vs. < 65 years)	0.597 (0.318–1.120)	0.108		
Tumor size (< 5 vs. ≥ 5 cm)	0.654 (0.351–1.220)	0.182		
Differentiation	0.604 (0.322–1.132)	0.115		
T stage (T1 + T2 vs. T3 + T4)	2.503 (1.245–5.030)	**0.010**	1.078 (0.319–3.651)	0.903
N stage (N0 + N1 vs. N2 + N3)	1.476 (0.791–2.753)	0.221		
Vascular invasion	0.891 (0.476–1.669)	0.719		
Lymph node metastasis	0.894 (0.481–1.663)	0.724		
Relapse	0.049 (0.017–0.144)	** < 0.001**	0.018 (0.005–0.066)	** < 0.001**
LAMC2 expression (Low vs. High)	2.500 (1.264–4.944)	**0.008**	7.316 (1.906–28.080)	**0.004**

### LAMC2 expression enhanced GC cell proliferation

**Fig. 3:**
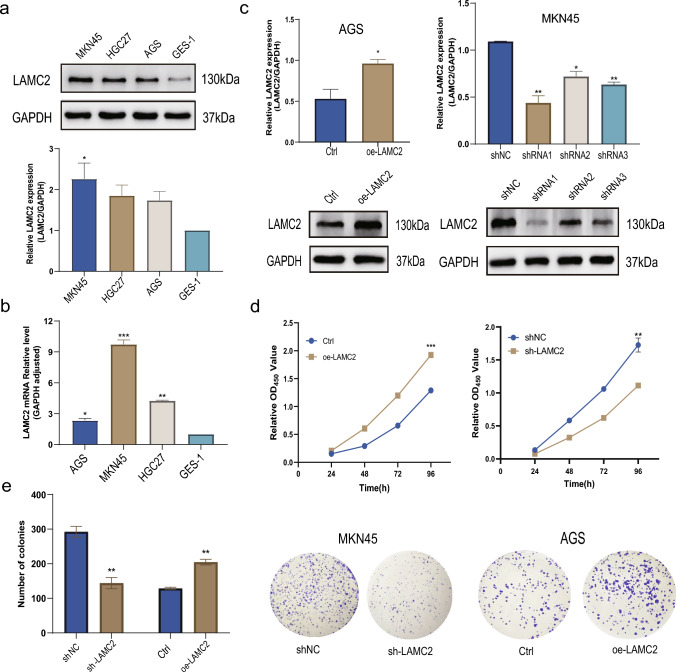
The expression of LAMC2 in GC cell lines and its impact on cell proliferation. **a** Western blot analysis shows that LAMC2 protein levels are higher in the three GC cell lines, HGC-27, AGS, MKN45, compared to the normal gastric mucosal cell line, GES-1.GAPDH was used as a loading control. **b** qPCR was used to assess LAMC2 mRNA expression in the cell lines. GAPDH was used as a loading control. **c** Western blot reveals transfection efficiency in LAMC2 protein levels following shRNA transfection. **d** CCK-8 and representative images of colonies in shRNA1-transfected MKN45 cells, over-expression vector-transfected AGS cells, and their respective control groups. The results are presented as the mean ± SEM from three independent experiments. (*p < 0.05; **p < 0.01; ***p < 0.001)

To assess the impact of LAMC2 on GC progression, LAMC2 expression in various GC cell lines was examined (Fig. 3a, b). In the findings, MKN45 cells displayed significantly elevated LAMC2 expression, whereas AGS cells exhibited comparatively reduced levels of LAMC2 (Fig. 3a, b). LAMC2 was knockdown in MKN45 cells via transfection with shRNA lentiviral vectors, while in parallel, LAMC2 was overexpressed in AGS cells using an overexpression lentiviral vector. The transfection efficiency was confirmed by immunoblotting analysis. To proceed with subsequent functional experiments, shRNA1 was selected, as it demonstrated the highest level of silencing efficiency (*p* < 0.01, Fig. 3c). To evaluate the impact of LAMC2 on the growth of GC cells, CCK-8 assays, and colony formation assays were conducted on MKN45 and AGS cell lines. In the CCK-8 assay, absorbance and graphs of cell growth curves were measured continuously for four days. Findings demonstrated that LAMC2 knockdown in the MKN45 cell line substantially decreased cell proliferation rate. Further, the growth vitality of the knockdown group was significantly lower. The effects of overexpression of LAMC2 on cell growth vitality and proliferation in the AGS cell line were also explored and demonstrated that LAMC2 overexpression significantly enhanced these cellular processes (*p* < 0.01, Fig. 3d, e).

### LAMC2 enhanced in vitro GC cell migration and invasion

**Fig. 4:**
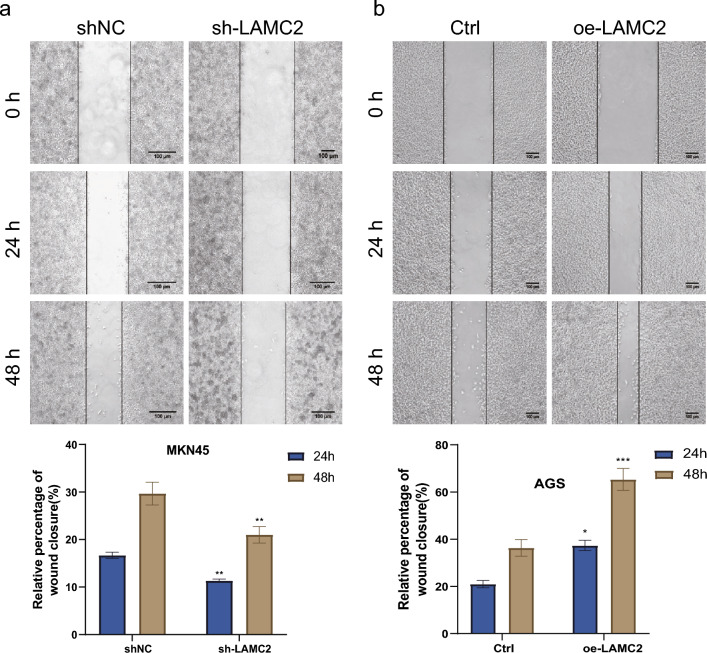
Cell scratch assays for the migration of GC cells. **a** The wound healing assay demonstrated that, at 24 h and 48 h, the gap between the wound edges was notably wider in LAMC2-silenced MKN45 cells compared to the control. **b** In LAMC2-overexpressing AGS cells, after 24 and 48 h, the gap between wound edges was significantly narrower than the control. The results are presented as the mean ± SEM from three independent experiments. (Scale = 100μm;*p < 0.05; **p < 0.01; ***p < 0.001)

**Fig. 5:**
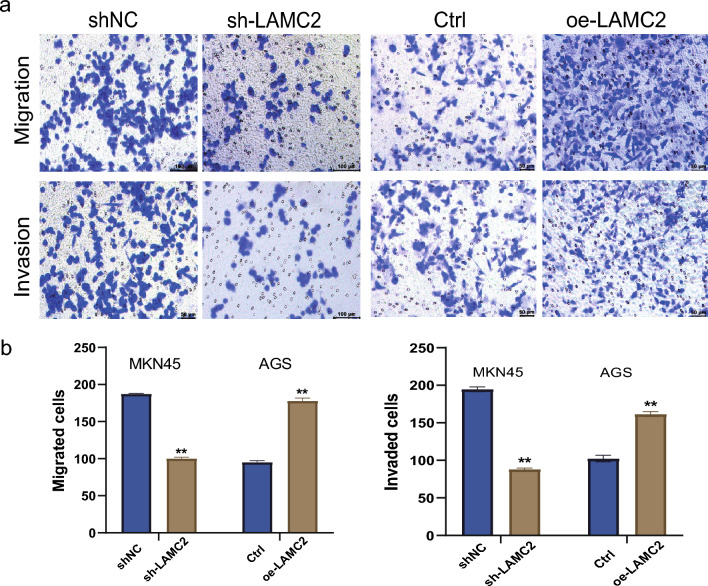
Transwell assays for the migration and invasion of GC cells. **a** Representative images demonstrate that migration and invasion are reduced in LAMC2-silenced MKN45 cells and increased in LAMC2-overexpressing AGS cells, compared with the corresponding controls. **b** Quantitative analysis of migrated and invaded cells. The results are presented as the mean ± SEM from three independent experiments. (Scale = 100μm;*p < 0.05; **p < 0.01; ***p < 0.001)

Findings from the GSEA revealed a significant link between the LAMC2 and the local invasion and metastasis. Besides, transwell and cell scratch assays were executed to examine the influence of LAMC2 on the invasive and migratory capacities of GC cells. The area of the scratch gap was evaluated at 0, 24, and 48 h post-viral infection via scratch assay analysis. Next, the impact of LAMC2 downregulation was noted on wound healing in MKN45 cells and revealed that the inhibition of LAMC2 remarkably reduced wound healing capacity, as evidenced by impaired cell migration (*p* < 0.01, Fig. 4a). Conversely, the upregulation of LAMC2 resulted in a significant increase in the migration of AGS cells. This observation suggests that LAMC2 overexpression actively promotes the migration of GC cells (*p* < 0.001, Fig. 4b). Transwell assay evaluated the proportion of cells exhibiting invasive and migratory properties at the 36h time point after viral infection. Findings demonstrated that LAMC2 downregulation in MKN45 cells substantially decreased the number of cells migrating the chambers and matrix gel, thereby suppressing cell invasion. In contrast, LAMC2 overexpression considerably enhanced the AGS cell numbers, migrating towards chambers, as determined by statistical analysis (*p* < 0.01, Fig. 5a, b).

### LAMC2 promotes in vivo GC cell proliferation

**Fig. 6:**
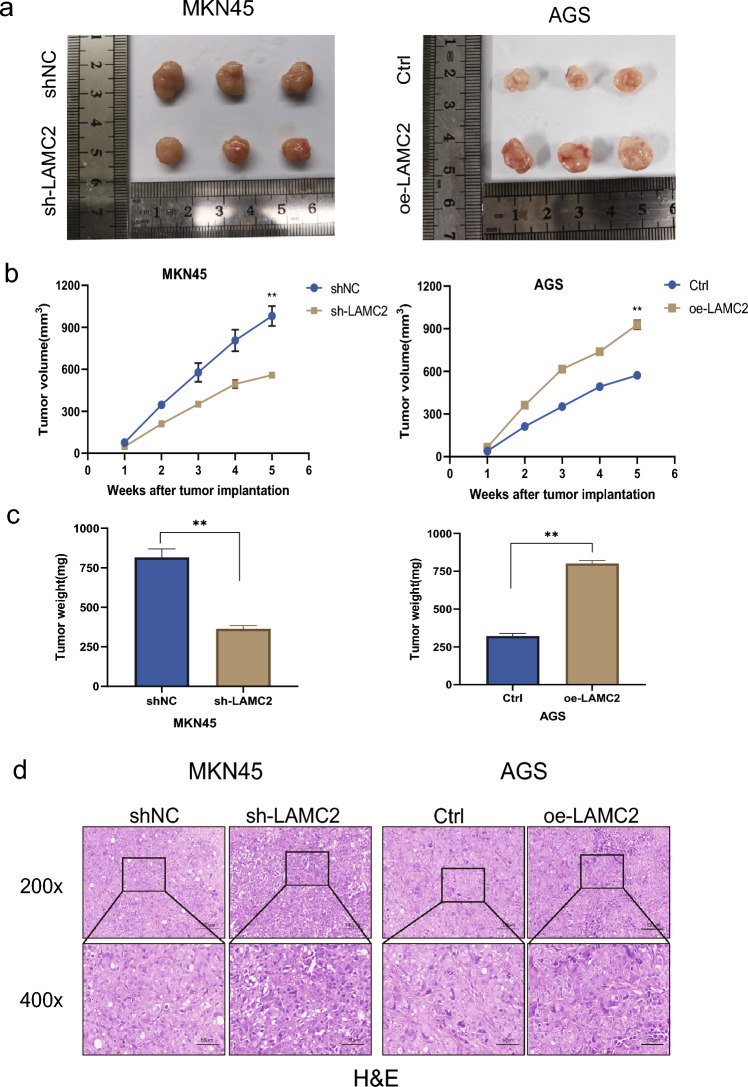
The xenograft tumor experiment demonstrates the proliferation of GC cells in vivo. **a** Images of subcutaneous xenografts from BALB/c nude mice.*N* = 3. **b** Tumor volume growth curves and **c** tumor weight changes of subcutaneous xenografts. **d** Representative images of H&E-stained tumor sections from nude mice injected with shRNA transfected cells. (scale: 100μm (top) and 50μm (bottom); *p < 0.05; **p < 0.01; ***p < 0.001)

**Fig. 7:**
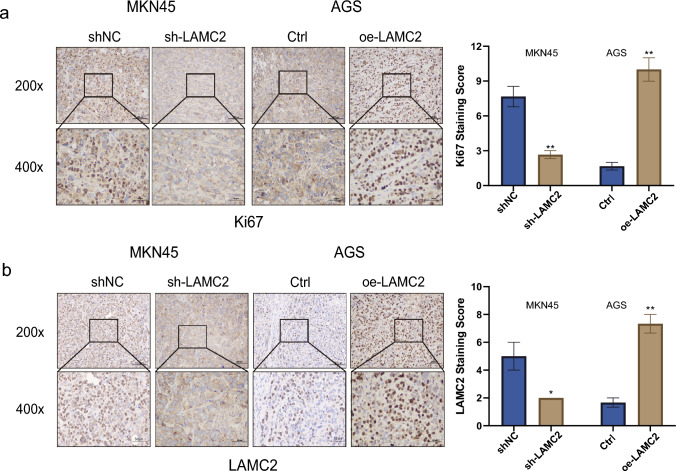
IHC detection of the expression of Ki-67 and LAMC2. **a** Expression of the proliferation marker Ki-67 in xenograft tumor tissue sections detected using IHC. **b** Expression of LAMC2 in xenograft tumor tissue sections detected using IHC. (Scale: 100μm (top) and 50μm (bottom); *p < 0.05; **p < 0.01; ***p < 0.001)

To elucidate the regulatory role of LAMC2 in GC progression in vivo settings, MKN45 cells were transfected with lentiviral vectors containing either control shNC or sh-LAMC2 sequences. AGS cells were transfected with pLenti-CDNA-LAMC2 and an empty vector. These transfections were performed to establish stable cell lines and subcutaneous tumor models in nude mice (Fig. 6a). Findings demonstrated that LAMC2 knockdown led to a substantially reduced tumor growth rate and volume compared to the control (*p* < 0.01, Fig. 6b, c). In contrast, LAMC2 overexpression substantially accelerated tumor growth, resulting in significantly larger tumor sizes than the control (*p* < 0.01, Fig. 6b, c). Figure 6d displayed the HE staining of consecutive tumor sections of the xenograft mouse model. In addition, Immunohistochemical analysis assessed Ki67 expression in the same model. The impact of LAMC2 knockdown was also investigated and revealed a notable decrease in Ki67 expression, indicating that the suppression of LAMC2 effectively reduced the capacity of tumor proliferation in the mouse model. Conversely, the overexpression group exhibited a contrasting trend (*p* < 0.01, Fig. 7a,b). Overall, these findings demonstrate that LAMC2 exerts a stimulatory effect on the in vivo growth and functions as a pivotal oncogene in the GC.

### LAMC2 induces EMT in GC cells by PI3K/Akt pathway

**Fig. 8:**
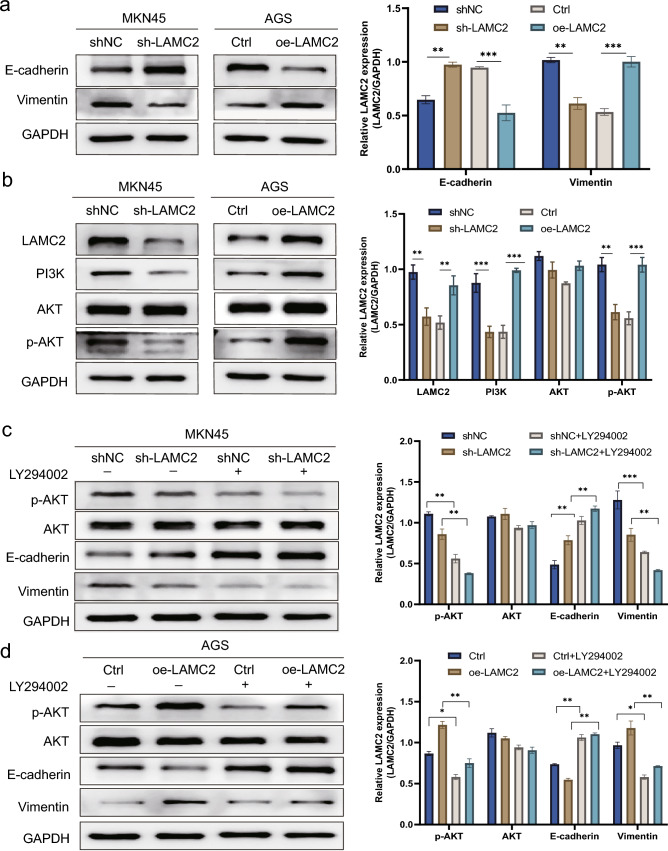
LAMC2 regulates PI3K-mediated Akt phosphorylation and EMT in GC cells. **a** Western blot analysis shows reduced N-cadherin and increased E-cadherin expression in LAMC2-silenced MKN45 cells compared with controls. Conversely, LAMC2-overexpressing AGS cells show increased N-cadherin and reduced E-cadherin expression compared with the controls. **b** Western blot analysis shows increased levels of PI3K, total Akt and reduced levels of p-Akt in the LAMC2-overexpressing AGS cells compared with the controls, whereas LAMC2-silenced MKN45 cells show reduced levels of PI3K, total Akt and increased levels of p-Akt compared with the controls. **c**, **d** The PI3K inhibitor LY294002 (10μM) suppresses PI3K signaling and reverses LAMC2-induced EMT, as analyzed by Western blot for protein levels. Each graph represents data from three independent experiments. (*p < 0.05; **p < 0.01; ***p < 0.001)

The transwell and scratch assay analyses revealed the involvement of LAMC2 in the GC cells. In light of the crucial significance of EMT in accelerating cancer cell invasion and metastasis, LAMC2 could potentially participate in the intricate molecular mechanisms of the EMT progression in GC cells. Western blotting investigated the expressions of EMT-related proteins in GC cells. Findings revealed that LAMC2 knockdown in GC cells led to an upregulation of E-cadherin and a downregulation of Vimentin. These alterations in marker expression suggest that knockdown LAMC2 could potentially impede the process of EMT. Conversely, the overexpression of LAMC2 enhanced the process of EMT (Fig. 8a). The involvement of the PI3K/Akt signaling pathway in tumor metastasis and invasion has been documented in the scientific literature. In this study, Western blotting examined the potential role of LAMC2 in advancing GC via its regulation of the PI3K/Akt signaling pathway. Findings revealed that the LAMC2 knockdown group substantially reduced the phosphorylation of PI3K and Akt. Additionally, there was an observable distinction in the total protein of Akt, which followed a similar pattern. In LAMC2 overexpression, the phosphorylation levels of PI3K and Akt exhibited a significant increase (Fig. 8b). The findings presented herein supported the involvement of LAMC2 in the initiation of the PI3K/Akt pathway within GC cells. To provide additional validation regarding the mediation of these effects via* a* specified pathway at the protein level, the cells were exposed to LY294002, an inhibitor targeting the PI3K/Akt pathway. As a result, the LAMC2 knockdown group exhibited a downregulation of p-Akt expression, resulting in an upregulation of E-cadherin and a downregulation of Vimentin levels when compared to the control (Fig. 8c). In the group of LAMC2 overexpression, treatment with LY294002 resulted in a remarkable reduction in the phosphorylation of Vimentin and Akt. Conversely, the levels of E-cadherin showed a substantial upregulation. The overall level of Akt was not significantly altered (Fig. 8d). The findings suggested the potential involvement of LAMC2 in the EMT process of GC cells, mediated by the PI3K/Akt pathway.

## Discussion

LAMC2 is a member of the laminin subfamily proteins contributing to adhesion and ECM. These proteins have been implicated in advancing different types of solid tumors (Gotte et al. 2018; Zhou et al. 2022). The clinical significance and role of LAMC2 in GC is not fully explored (Rousselle et al. 2020). The current study elucidated the level of LAMC2 in GC and investigated the clinical implications of this molecular target in GC. Findings demonstrated a noteworthy upregulation of LAMC2 in GC tissues compared to adjacent tissues. Moreover, GEO and TCGA datasets analysis further support a substantial association between LAMC2 overexpression and the incidence of tumors by TNM staging in GC. The study analyzed expression patterns of LAMC2 in GC cell lines exhibiting varying degrees of differentiation by qRT-PCR and Western blotting. The outcomes revealed that LAMC2 showed increased levels not only at the RNA level but also at the protein level. Significantly, our research revealed that LAMC2 has the potential to function as a standalone predictive biomarker for DFS in individuals diagnosed with GC. Despite the limited sample size of 70 patients with stomach cancer in the present retrospective investigation, our initial findings serve as a foundation for forthcoming prospective studies. These findings indicated a correlation between the upregulation of LAMC2 expression and the onset of GC.

Recent investigations have analyzed that the LAMC2 is increased in multiple types of cancer and is involved in promoting the advancement of tumors (Erice et al. 2023; Ferreira et al. 2022; Fu et al. 2022). Besides, to investigate the biological functions of LAMC2 in GC, stable LAMC2 knockdown and overexpression cell lines were generated. The findings presented that the overexpression of LAMC2 remarkably enhances GC cells' proliferation, invasion, and tumorigenic capacity. Conversely, the suppression of LAMC2 expression effectively counteracts these biological processes. Additionally, the overexpression of LAMC2 has been observed to induce EMT, consequently improving tumor cells' invasive and migratory capacities. The findings also provide evidence supporting the potential involvement of LAMC2 in GC pathogenesis, thereby highlighting its prospective use as a novel therapeutic target.

The PI3K/Akt signaling is widely recognized as cancer's most common disrupted pathway. It is commonly hyperactivated in various malignant tumors, such as GC, and is crucial to facilitating aggressive tumor growth and proliferation (Chai et al. 2018; Zhu et al. 2020). Previously documented that TTPAL induces initiation of the PI3K/Akt signaling pathway via interaction with NNMT, consequently facilitating the growth, migration, and penetration of GC cells (Liu et al. 2021). In addition, long non-coding RNA (lncRNA) THAP7-AS1/CUL4B exerts a stimulatory effect on the GC cell invasion and development by the PI3K/Akt signaling pathway (Liu et al. 2022). The findings suggested that the PI3K/Akt signaling pathway substantially influences the malignant biological characteristics of GC.

Previous analyses have demonstrated that the Laminin family of adhesive proteins displays a crucial influence on the protein network regulating the equilibrium of the PI3K/Akt signaling pathway (Hsu et al. 2020; Liang et al. 2018). Western blot analysis exhibited the potential impact of LAMC2 downregulation on the PI3K/Akt signaling pathway. Knockdown of LAMC2 resulted in a remarkable reduction in the phosphorylation of Akt, while the total levels of Akt protein remained unaltered. Conversely, the overexpression of LAMC2 facilitated the activation of this signaling pathway. Findings also suggested that LAMC2 is crucially involved in facilitating tumor invasion in GC via activation of the Akt signaling pathway. EMT is a biological process characterized by losing polarity and adhesion in epithelial cells. This process occurs due to molecular reprogramming and subsequent phenotypic alterations, ultimately acquiring migratory and invasive properties similar to mesenchymal stem cells (MSCs) (Li et al. 2023; Yu et al. 2022). Western blot analysis detected expression of EMT markers, Vimentin, and E-cadherin. Vimentin, a prominent constituent of the intermediate filament, exhibits selective expression in healthy MSCs, while its expression is significantly upregulated in different cancer types. E-cadherin, a transmembrane glycoprotein, is pivotal in forming and maintaining adhesive junctions, facilitating cell adhesion, and preserving the epithelial phenotype (Babaei et al. 2021; Huang et al. 2022). As a result, the knocking down of LAMC2 led to a reduction of Vimentin and an elevation of E-cadherin in GC cells. These findings suggest that the EMT process was impeded. Conversely, LAMC2 overexpression exhibited contrasting effects, indicating an increase in EMT.

Subsequently, LY294002, a potent inhibitor targeting the PI3K/Akt pathway (Akinleye et al. 2013), was used. The outcomes revealed that LY294002 exhibited the capability to reverse the EMT changes and indicated that the Akt signaling activation participates in the EMT process mediated by LAMC2. The findings further suggested that the invasion and migration facilitated by LAMC2 are potentially mediated via the PI3K/Akt pathway. A mouse tumor xenograft model has been developed to explore the action of LAMC2 in controlling the metastasis of GC. Further research is warranted to determine whether the signaling pathway is activated by LAMC2 and is involved in the interactions with crucial downstream genes of PI3K. Henceforth, LAMC2 can potentially facilitate GC cell proliferation and EMT, which is intricately linked to the activation of the PI3K/Akt pathway. However, studying potential factor-related interactions through which LAMC2 may contribute to the GC progression could be a prospective area of research.

## Supplementary Information

Below is the link to the electronic supplementary material.

Supplementary file1 (PDF 153 KB)

## Data Availability

All data generated or analyzed during this study are included in this article. The original data can be provided by the corresponding author upon reasonable request.
