# Supplementary figures and images for: LAMC2 regulates the proliferation, invasion, and metastasis of gastric cancer via PI3K/Akt signaling pathway
Source: J Cancer Res Clin Oncol. 2024 May 4;150(5):230. doi: 10.1007/s00432-024-05720-7 (PMC11069487; doi:10.1007/s00432-024-05720-7)

**Figure.S1**

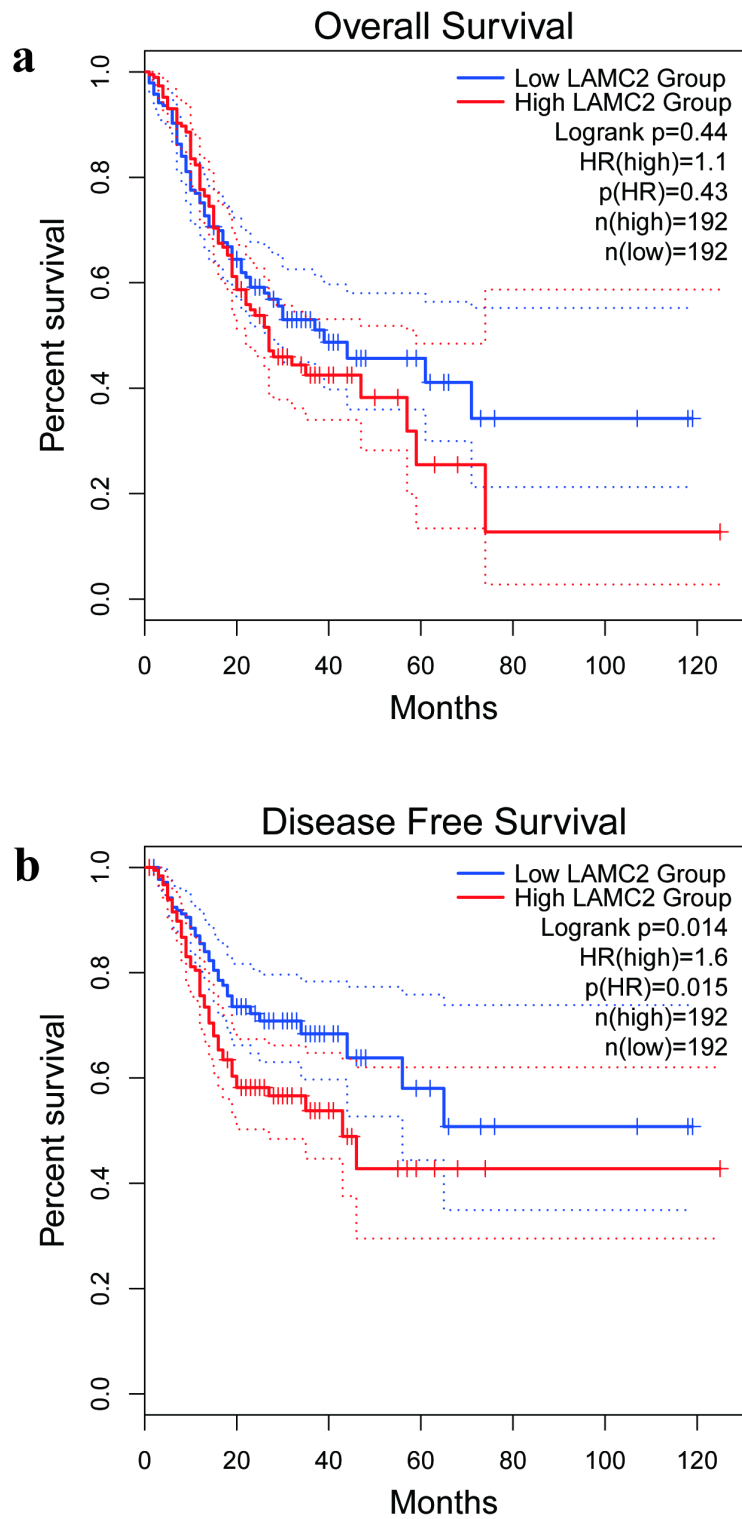

Supplement: Supplementary file 1 — Supplementary file1 (PDF 153 KB) [file 432_2024_5720_MOESM1_ESM.pdf]
